# Exploring ambulance clinicians’ clinical reasoning when training mass casualty incidents using virtual reality: a qualitative study

**DOI:** 10.1186/s13049-024-01255-5

**Published:** 2024-09-16

**Authors:** S. Heldring, V. Lindström, M. Jirwe, J. Wihlborg

**Affiliations:** 1 Department of Health Promoting Science, Sophiahemmet University, Lindstedtsvägen 8, Box 5605, 114 86 Stockholm, Sweden; 2 AISAB Ambulance Service, Johanneshov, Region of Stockholm Sweden; 3 Division of Ambulance Service, Department of Nursing, Umeå University, 901 87 Umeå, Region of Västerbotten Sweden; 4 Department of Health Sciences, Swedish Red Cross University, Box 1059, 141 21 Huddinge, Stockholm, Sweden; 5 Department of Neurobiology, Care Sciences and Society, Karolinska Institutet, Solna, Sweden; 6 School of Health and Welfare, Dalarna University, 791 88 Falun, Sweden

**Keywords:** Ambulance services, Chart-stimulated recall technique, Clinical reasoning, Disaster preparedness, High-fidelity simulation, Mass casualty incident, Virtual reality

## Abstract

**Background:**

How ambulance clinicians (ACs) handle a mass casualty incident (MCI) is essential for the suffered, but the training and learning for the ACs are sparse and they don’t have the possibility to learn without realistic simulation training. In addition, it is unclear what type of dilemmas ACs process in their clinical reasoning during an MCI. With virtual reality (VR) simulation, the ACs clinical reasoning can be explored in a systematic way. Therefore, the objective was to explore ambulance clinicians’ clinical reasoning when simulating a mass casualty incident using virtual reality.

**Methods:**

This study was conducted as an explorative interview study design using chart- stimulated recall technique for data collection. A qualitative content analysis was done, using the clinical reasoning cycle as a deductive matrix. A high-fidelity VR simulation with MCI scenarios was used and participants eligible for inclusion were 11 senior ACs.

**Results/conclusion:**

All phases of the clinical reasoning cycle were found to be reflected upon by the participants during the interviews, however with a varying richness of analytic reflectivity. Non-analytic reasoning predominated when work tasks followed specific clinical guidelines, but analytical reasoning appeared when the guidelines were unclear or non-existent. Using VR simulation led to training and reflection on action in a safe and systematic way and increased self-awareness amongst the ACs regarding their preparedness for MCIs. This study increases knowledge both regarding ACs clinical reasoning in MCIs, and insights regarding the use of VR for simulation training.

**Supplementary Information:**

The online version contains supplementary material available at 10.1186/s13049-024-01255-5.

## Background

Preparedness for mass casualty incidents (MCI) is increasingly in focus for healthcare professionals following conflicts and terrorist threats worldwide. One of the healthcare organisations that must be prepared is the ambulance service, which arrives early at the scene of an incident. Around the world, ambulances are staffed by ambulance clinicians (ACs) of diverse educational backgrounds [1, 2]. No matter what experience or educational background, working as an AC requires being prepared to act in an MCI [3]. ACs’ duties in an MCI include different kinds of decision-making, triage, assessments, and treatment of the injured. The work in an MCI also includes problem-solving and acting on safety and security matters. ACs need to be prepared but must also be flexible and open-minded when caring for the injured and are expected to maintain control and be efficient [3]. A way to achieve and cultivate the necessary abilities to act in an MCI is through learning by experience and reflection on action [4]. This can be performed as a four-phase spiral of learning, where the first phase is a concrete experience, followed by observation of and reflection on the experience, then drawing a conclusion based upon the reflections, and last, testing and repeating as Kolb described [4]. However, even if all four phases are included in a learning activity, the ability to use the gained experiences is not granted. The learner can end up being strengthened but relatively unchanged, or the learner can change and become transformed by the learning experience [5]. A well-developed reflection on action allows learners to reflect on experiences and enables them to abstract and transfer their learning into new contexts [6]. Becoming aware of undesired behaviours and thereby being able to correct them is an important reason to perform reflection on action [7].

One way to learn by experience that provides an easily accessible reflection on action is high-fidelity virtual reality (VR) simulations, which is an increasingly popular technique and is trending in learning environments worldwide [8–10]. VR simulation, where the ACs can repeatedly step into a virtual world and interact and be exposed to realistic and stressful situations, has the potential to develop knowledge, skills, and experience [9]. Simulating an MCI using VR is a way to use experiential learning for knowledge development [4]. There is a concrete experience when performing the VR simulation, and it is possible to observe the experience afterward by looking at the video record of the simulation and reflecting on it. Conclusions can be made from those reflections, and the experience can be tested and repeated by redoing the VR simulation, which ideally will lead to a change in behaviour [5].

**Fig. 1:**
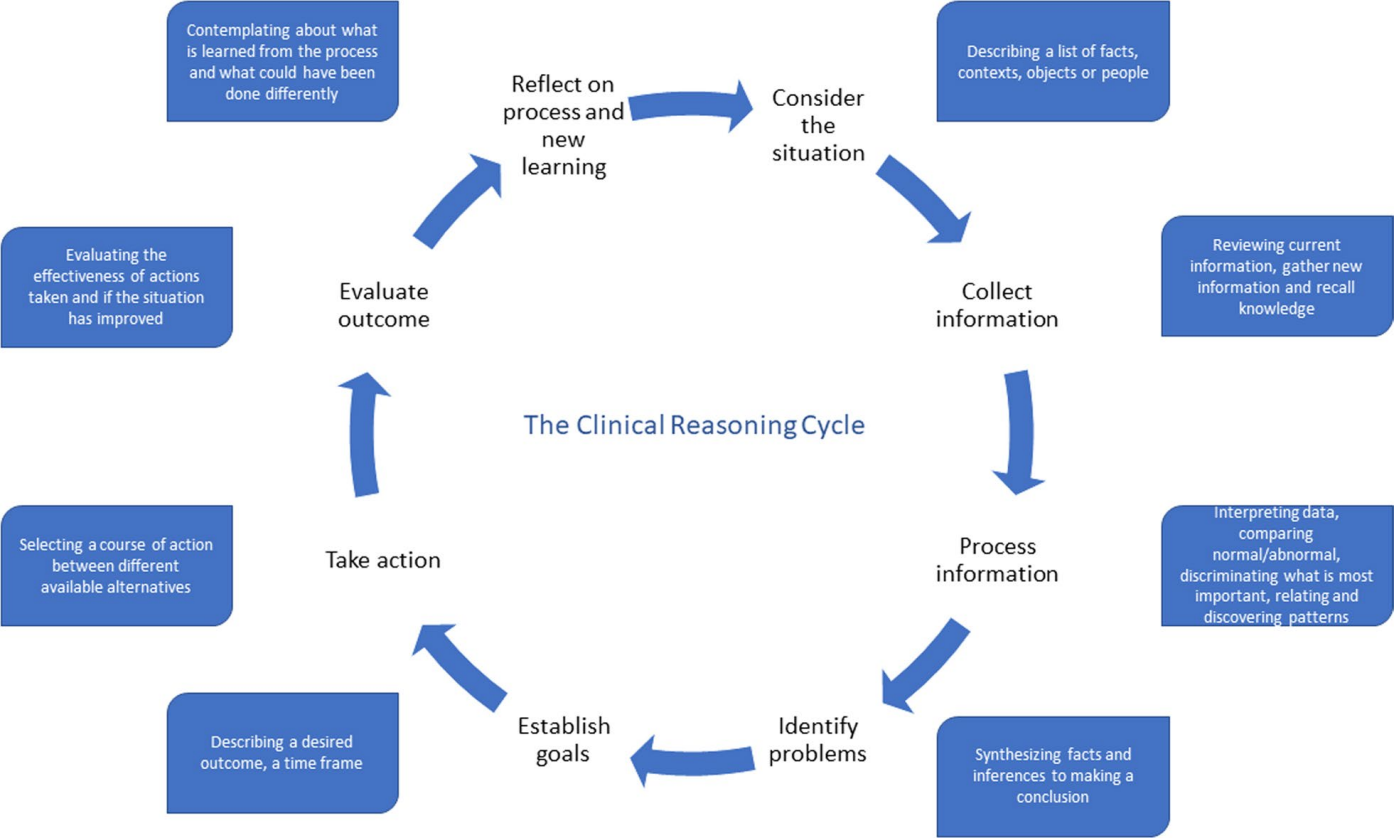
The clinical reasoning cycle [11]

VR simulation allows research on subjects with little or no accessibility in clinical environments, as it allows the possibility to repeat and record scenarios while not exposing patients to harm. A subject that is vital to study is how ACs practice clinical reasoning in an MCI situation. Clinical reasoning is a process where clinicians consider the situation, collect information, process the information, identify problems, establish goals, take actions, evaluate outcomes, and reflect on the process and new learning [11] (Fig. 1).

Clinical reasoning is an important competence in an MCI, as it enhances the capacity to differentiate between a clinical problem that needs urgent attention and one that is less urgent [11, 12]. Tanner [13] defines clinical reasoning as the process of making clinical decisions by selecting from alternatives, weighing evidence, using intuition, and pattern recognition. The cognitive thinking strategies used in decision-making and clinical reasoning have not been extensively studied, yet differences between novices and seniors are described [14]. Seniors tend to collect more cues from a broader range than novices and are generally better at predicting what may happen next in a way that seems automatic or instinctive [14, 15]. In contrast, novices tend to be more reactive, searching for cues after already identifying a problem [14]. This could lead to patient safety issues in MCIs if novices detect problems later than seniors. Another aspect is that seniors tend to use non-analytic reasoning as they are in a position where they do not need to reason because they rely on their intuition [15]. However, more analytical reasoning can be achieved if a conscious effort is made to carefully consider all evidence available before generating a hypothesis [15]. Structured use of the clinical reasoning process in teaching is appropriate when training and learning to manage complex clinical situations and has an impact on clinical practice [11]. The optimal form of clinical reasoning is a model in which both analytic and non-analytic processes play a role [15].

ACs need to be prepared to handle an MCI, and they need training and learning, which can be achieved with high-fidelity VR simulation. Clinical reasoning is an important competence and vital for decision-making but there is a lack of knowledge on how ACs’ clinical reasoning is performed in MCIs. With VR simulation, the ACs’ clinical reasoning can be explored in a systematic way. Therefore, the objective of this study was to describe ACs’ clinical reasoning when simulating an MCI using VR.

## Methods

This study used an explorative interview design using the chart-stimulated recall technique [16] for data collection. Data were analysed using deductive qualitative content analysis following Elo Kyngäs [17]. The study was conducted and reported in accordance with the consolidated criteria for reporting qualitative research (COREQ) [18] (Online appendix 1).

### Settings

Data were collected in Stockholm, Sweden, from March 2021 to December 2021. A high-fidelity VR simulation with MCI scenarios, named GoSaveThem® [19] was used. GoSaveThem® was invented by a local AC with an interest in MCIs, VR, and education. This software was selected because it was the only known available application adjusted to Swedish conditions, and we wanted the setup to be as true to the real life-world for the participants as possible. In the VR simulation, the participants were directed to perform primary triage, a system to initially sort out whom to help first, intending to save as many injured as possible [20, 21].

The VR simulation was performed with a VR system from HTC VIVE®. The glasses enabled a fully immersive head-mounted display making it possible for the participants to walk around in the virtual world and interact with the suffering and injured. The handheld controllers gave vibrational feedback, when the participant pressed the artery of an avatar patient in the VR scenario, making it feel like assessing the pulse of a real patient. Other actions available with the handheld controllers were walking, applying tourniquets, using a torch, looking at a watch, viewing the primary triage guidelines whenever needed, and deciding the level of primary triage. The participants could also listen to the sound of breathing when holding their head close to the avatar patient’s chest, enabling them to assess the respiratory rate in a realistic way.

### Scenario

The VR simulation took place at the workplace of the participants, with a researcher and the participant present in the room. First, the participants familiarised themselves with the VR technique, which generally took about 5–10 min. Then they received information about the ambulance assignment and were asked how they wanted to prepare themselves on the way to an MCI. When starting the MCI scenario, the participants found themselves located outside their ambulance on the street next to a subway station entrance. They could see several people running away, and some injured patients standing on the pavement. A police officer was telling the participant that it was safe enough to enter the subway station. The scene that faced the participants was a chaotic site after an explosion. Down the stairs to the subway station, there was a train standing by the platform and a lot of bricks and dust on the ground. Injured people, crying and screaming for help, were lying everywhere. In the farthest train carriage, the light was out, but on searching through the carriage the participant could find an open bag with unexploded explosives. The MCI scenario ended either when the participant decided to evacuate after finding explosives, or after all the injured were assessed and had received their primary triage. The time spent on the scenario before ending the simulation was 6:54–23:53 min (m = 16.5/md = 17).

### Data collection

Directly after the VR simulation, the participant filled in a demographic questionnaire with questions on age; gender; professional title; years of employment in the ambulance services; previous experience of primary triage; previous experience with MCIs; and previous experience using VR.

When the questionnaire was completed, the participant was seated in front of the computer with the video recording of the VR simulation and was interviewed by the first author while watching the video. The time passing between the VR simulation and the start of the interview was between 4 and 11 min. The chart-stimulated recall technique [16] was used during the interview, as the technique helps participants to describe their clinical reasoning; the participants were asked to pause the video recording of their VR simulation when identifying themselves taking decisions and were encouraged to think aloud when describing their clinical reasoning. Video playback was paused by the participants for clinical reasoning reflections between 14 and 23 times. The researcher used a semi-structured interview technique, to help the participant to articulate their clinical reasoning and decision-making. Questions used were: What did you see, think, and feel? What made you make this decision and why? Can you describe what happened? What did you expect to happen? What are your thoughts regarding this? Can you explain more? Can you give examples? Is there anything you wished you had done differently? and What do you think could have happened if you had taken another decision? The interviews (n = 11) were audio-recorded with a smartphone on flight mode and transcribed verbatim. After the transcription, the audio records were placed in a secure location, only available to the researchers. The interviews lasted between 28 and 57 min (m = 39.7/md = 40).

### Participants

Convenience sampling strategies were used. Informational posters and emails with information on the study were distributed to invite all employed ACs (n = 42) who worked in special ambulance units, which are targeted to support and command major incidents, such as MCIs. These ACs were easy to access as the special ambulance units were on standby for a substantial time during their working hours and were thereby available for simulation and interview on duty.

Participants eligible for inclusion were 11 ACs, and all were given an individually scheduled time for the VR simulation and interview. There were no dropouts. The same type of clinical reasoning appeared after about eight interviews, and as the data seemed to be saturated, we stopped the efforts to reach more participants than those already accepted to participate. The ACs in these special ambulance units are supposed to be senior and have theoretical and practical education to lead the work at major incidents and are supposed to help other ambulances with structure and organisation in MCI situations. Nine of the ACs were master’s degree nurses and two were emergency medical technicians (EMTs). Their length of experience as ACs ranged between 6 and 18 years (m = 10,1/md = 9), and all the nurses also had clinical working experience before starting as ACs. The ages of the participants ranged between 33 and 56 years (m = 41.2/md = 40) and the majority were male (n = 7).

### Data analysis

Data were analysed using deductive qualitative content analysis [17]. The theoretical framework and conceptual model that was used in the analysis, was a clinical reasoning cycle with eight phases, in accordance with Levett-Jones’ description [11]. The data analysis started by transcribing the interviews and then all interviews were read through to get a sense of the whole. Meaning units relevant to the study aim were identified, condensed, and coded. A structured categorisation matrix of the eight phases of the clinical reasoning process as categories were developed [11]. The condensed meaning units were gathered and allocated by clinical reasoning content to each category. Then, the data with similar content were grouped, abstracted, and interpreted to form the result. During the analysis process, comparisons with the original transcribed interviews were performed and discussed among the authors to agree on the final results.

### Ethical considerations

The study was conducted aiming for good ethical practice with transparency, accuracy, and avoidance of plagiarism [22] and aligned with the Declaration of Helsinki statement of ethical principles for medical research [23]. All participants signed a consent form and were willing to contribute to this study without any coercion. The participants had the right to withdraw from the study at any time and were informed that participation/refusing to participate was completely voluntary and would have no effect on their employment. Ethical approval was sent to the Swedish Ethical Review Authority before conducting the study, and they answered with an advisory opinion that Swedish law does not require a formal ethics review for this kind of study (dnr: 2019-00697).

## Results

**Fig. 2:**
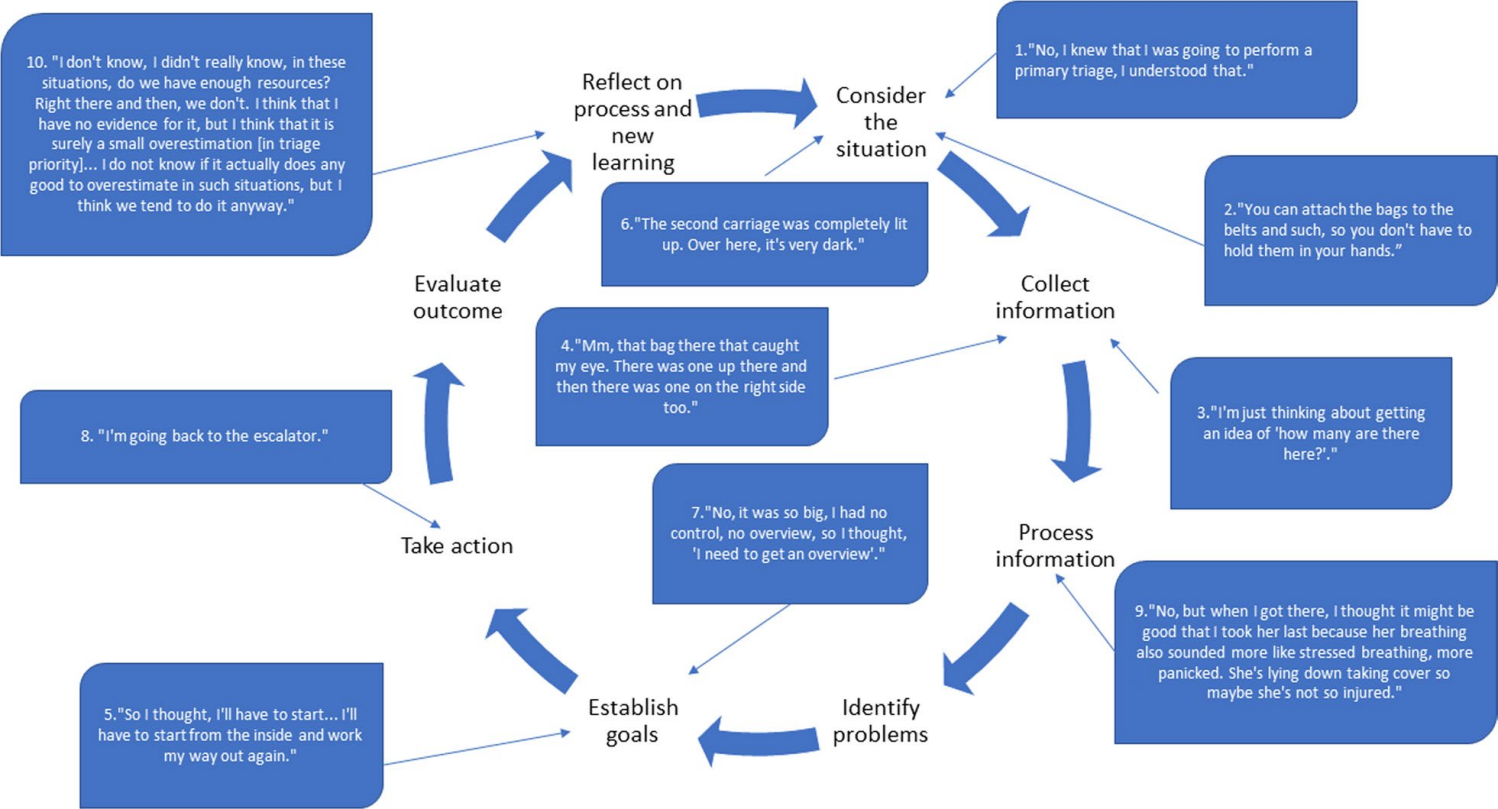
The clinical reasoning cycle of a participant with several processes ongoing at the same time. Nr 1–10 is showing the actual order of clinical reasoning quotes, according to the timeline in the interview (participant #10)

All phases of the clinical reasoning cycle were found to be reflected upon by the participants during the interviews, showing a variation between analytic and non-analytic reasoning. The clinical reasoning among the participants almost never appeared as an ideal cyclical process about one patient or situation; instead, several processes were ongoing at the same time, and the clinical reasoning cycle was often cut short and repeated during the interviews (Fig. 2). The result below is presented in the order of the clinical reasoning cycle, not according to the VR simulation timeline.

### Consider the situation

To consider the situation, the participants expressed the need to form a general impression of the scene. They described the lack of overview they wanted of the scenario initially and how they repeatedly re-evaluated their impression of the chaotic situation as the scenario went on. To think aloud about the situation was said to be a strategy for keeping control. They reminded themselves of what equipment to use, the rules of leadership, and the guidelines to follow. They considered the explosion in the subway station to be a possible act of terrorism which raised their awareness of other threats in the area.

When interpreting the scenario as an MCI, they started to prepare themselves for the types of typical injuries to expect patients to suffer when there has been an explosion. They expressed a need to estimate whether the available resources were enough to handle the situation.

I thought that, in the alarm call, this was a suspected special event and that there would be many injured at the scene. That we would probably need many resources. I also thought, what has exploded, are there possibly any dangerous substances here? (participant #9)

### Collect information

Collecting information and assessing the scenario was described to be challenging as it was difficult to separate relevant information in the large flow of impressions and feelings. The participants expressed intentions to be active in collecting information when visualising the scene to identify safety and security issues. They described how they identified potential threats of additional explosions, collapsing walls/roofs, and poisonous smoke. They expressed a desire to collect further information to be able to proceed and, searched for collaboration partners. To re-evaluate the situation constantly was said to be of importance and they described how they recalled previous knowledge to make sure their assessments of the scenario were plausible.

I looked around. I see victims, one, two, three, one lying outside the subway barriers, I look up at the ceiling to see if there is anything dangerous hanging there. (participant #5)

The participants expressed the intention only to collect information about the patients to enable primary triage because conducting excessively rigorous assessments made them focus too much on one patient. However, expressions of uncertainty about their own assessments were found, as when they described difficulties in evaluating vital signs, especially in children.

### Process information

The participants tried to process and discriminate what contextual information was important in the large flow of impressions. They described how they used their previous knowledge, tried to group information to anticipate potential outcomes, and questioned whether the information they had collected was reliable. Both subjective and objective cues were taken into consideration when they described recalling their intuition and past experiences.

When processing information about the patients, they mainly interpreted vital signs and typical injuries after explosions. These interpretations were often brief and were performed in a non-analytical way. The decision on primary triage levels was based on a more analytical reasoning process, as they discussed whether they should deviate from the primary triage guidelines and base their clinical decision on intuition or types of injuries instead. They reasoned about the essence of intuition and referred to previous experiences and theoretical knowledge. Generally, they preferred to give higher priority levels than lower ones, after they had processed the collected information about the injured.

The child couldn’t walk but screamed, so I thought that it wasn’t that bad. It was very difficult to hear the respiratory rate because she was screaming, so I decided to skip the breathing and check the pulse, which I found to be normal. (participant #3)

### Identify problems

When the participants described identifying problems in the context, they drew conclusions about collaboration, safety and security issues, communication, and organisation. The participants tended to be quick in identifying what the patient may be suffering from. However, when they described conclusions made about primary triage the reasoning became more expanded.

It is actually wrong, but I based my assessment on the type of injury even though it wasn’t a primary triage parameter, and I classified her as red [highest priority] because she could soon get worse. (participant #3)

### Establish goals

When establishing goals for strategies of how to work, the goals were often task-oriented. The goals established were about mental strategies and own actions. The participants described goals for patient care and time consumption but not for individual patient outcomes. However, when describing setting goals for more than one patient, goals for the outcome existed, for example, to save as many as possible. They also reasoned about goals connected to primary triage guidelines and clarified how they personally planned to act upon them.

I have a strategy. First, check that it is safe, and then I will proceed in a certain order; otherwise, I will lose track of whom I have attended to. (participant #2)

### Take action

At the beginning of the scenario, actions were frequently described as actionable grabbing bags with different kinds of equipment, but there were also decisions taken to bring only what was needed for their pre-decided tasks, as it is easier to move more quickly if there is not too much to carry.

The participants acted to get a better view and understanding of the scenario. Actions were also described as connected to time as they intended to act quickly and efficiently to save as many as possible. Actions regarding safety and security were constantly ongoing. They described, for instance how they were checking bags lying around, searching through subway carriages, and deciding to evacuate when detecting a severe security threat.

When taking action for patient care, there were descriptions of assessments or interventions carried out but also of decisions taken not to act. The interventions were mainly concerned with basic life support. Actions described to decide the priority level in the triage were performed according to guidelines but also depended on the types of injury and patient behaviour. Moreover, the participants repeatedly counted the number of patients and described how they acted to keep track of that number and how they lacked the possibility to record it with a pen and paper.

I went first to the one who was closest. (participant #2)

The participants made communication initiatives and described how they talked to patients, even if they did not get any answers. They also said they tried to collaborate with police officers in the scenario and simulated radio communication.

### Evaluate action

Evaluations were described regarding how they acted when proceeding in the scenario, and how strategic decisions were taken accordingly. Dissatisfaction with wasted time and not acting efficiently was expressed. There were also expressions of self-criticism regarding how they formed an opinion of the scenario, such as security awareness and their own safety behaviour.

Something I could have done differently is to have a different scanning approach. I have to think in 3D; I can’t think in flat terms. (participant #1)

The participants evaluated the patient care briefly. They were partly self-critical regarding actions of care when it came to interventions, assessments, and how they decided to prioritise among the patients. They described evaluating the outcome when interventions were made but also when the decision was not to act. They emphasised how important communication was and pointed out things they communicated well but also when they wished to improve their own communication with patients and collaboration partners. They also evaluated their simulated communication on the radio as improvable.

### Reflect on the process and new learning

Detailed reflections were described during this last phase of the clinical reasoning cycle. The participants critically reasoned about their systematic work strategies and declared that time frames and guidelines are supposed to be followed but they detected flaws in their own ability to follow them. There were descriptions of doubt and remorsefulness at how they decided on priorities based on the patient’s age, injuries, and uncertainty about vital signs. Even though primary triage guidelines were pre-determined to be used, they critically reviewed how well these guidelines led to best practices. At the same time, they stressed the importance of having distinct instructions and guidelines that are easy to follow.

When reasoning about their patient care actions, they argued about the need to move on and be effective but at the same time, they accepted that it was important to recognise patient needs and to avoid making mistakes because of rushing. They described a negative emotional aspect of leaving patients behind when they had to proceed with other patients, especially when the patient they left was a child. They also concluded that MCIs are demanding, and not comparable to taking care of one patient at a time. They reasoned that they preferred triaging with too high priority rather than too low, but ethical concerns were raised that this type of decision may increase morbidity and mortality. Furthermore, they reflected upon the allocation of resources and who was prioritised for transport to the hospital. They reasoned that the workload should decrease as soon as other personnel started to arrive at the scene.

When reflecting on communication they believed talking would have required a greater effort in a real-life scenario, no matter if it was with patients, collaboration partners, or on the radio. Training on how to communicate better was described to be important, and they said that self-reflecting on this scenario taught them more about their personal way of communicating.

I would have liked to talk to everyone really, whether they can hear me or not, just to say that help is coming soon, you will be taken care of. (participant #1)

**Fig. 3:**
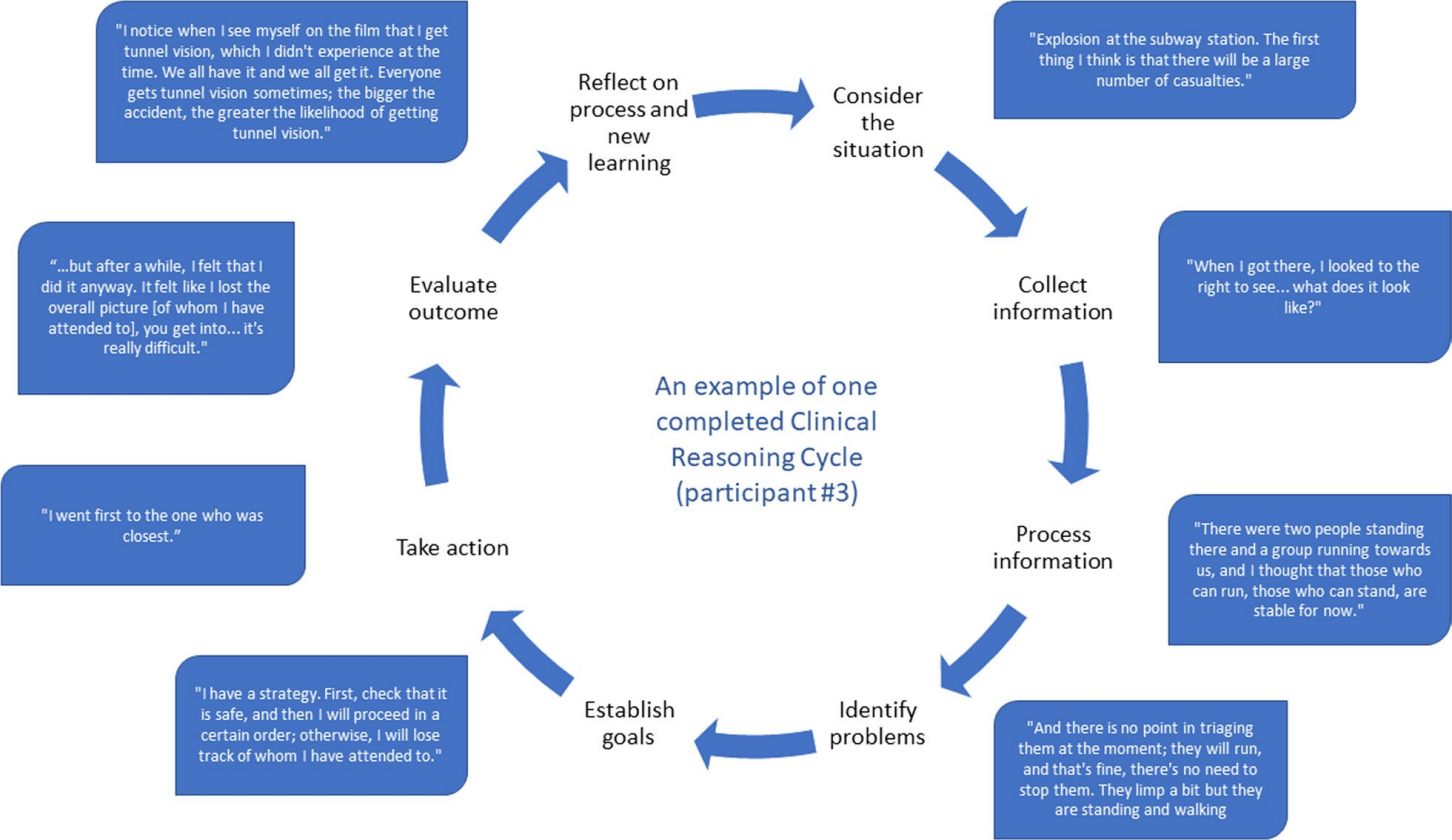
The clinical reasoning cycle [11]

The participants described lacking intuition when they were in the scenario and were convinced that repetitive training would lead to better intuitive actions. Increased awareness of how important it is to actively get a visual overview was mentioned, as this assisted in maintaining control and being able to make the right decision at the right time. When viewing the video recording, there were descriptions of having tunnel vision during the simulation, with decreased peripheral vision and limited analytical thinking (Fig. 3). This led to reflections about real-life scenarios and how they now had identified some of their own behaviour regarding stress and tunnel vision, which was described as valuable learning. Managing stress by being systematic, disciplined, and calm, was believed to reduce the risk of losing control.

And if you get to practice more, then you can see if you perceive things differently, do things differently. That would be great. Because you get to test yourself; I have already found things I need to work on. (participant #2)

When reasoning about risk assessments, the participants believed they would have put more emphasis on personal safety and security in a real-life scenario. They critically reviewed the kinds of risks one is willing to take on the duty, and the importance of reflecting on this matter beforehand to become more prepared. To be able to rely on the security assessments made by collaboration partners, like fire brigades and police officers, was found to be crucial. At the same time, they expressed the importance of being somehow sceptical, and aware that collaboration partners could have missed something due to a heavy workload. Furthermore, they also reflected on what roles one has in comparison to different types of collaboration partners and the importance of cooperating and helping each other, as a team.

The participants said that they wanted to find strategies to work more analytically in the future, using reflection to alter self-image, awareness, and behaviour. Even after completing this VR scenario, they said they were not completely confident about how they would react in a real-life scenario and expressed a desire to become more prepared through more training and learning. They declared that they would be troubled if they could not meet their own expectations in a real-life MCI and made high demands on themselves to act rationally, no matter the surroundings. Reflections about themselves also included their personal responsibility to act as a professional and human being and the importance of being mentally prepared.

## Discussion

In accordance with the theoretical framework [11], the participating ACs in the study described their clinical reasoning when handling an MCI during a VR scenario. The participants tended to use non-analytic reasoning regarding the work tasks included in the guidelines, such as performing patient assessments and giving first aid to the patients. Acting on these kinds of tasks seemed to be almost automatic, and the reason for this could be related to the fact that all participants in this study were seniors in their profession and knew what to do and how to do it [15]. However, when the tasks involved decision-making with no clear guidelines, the reasoning was more analytic, and they gathered information from a broader range, as can be expected when understanding clinical reasoning theories [14, 15]. One example of such complex reasoning was about safety and security matters. Their reasoning showed that even if they mostly detected the same safety and security threats and described similar mental strategies, they had several different approaches to acting upon them. However, they agreed that it was important to have thought through and decided upon safety and security strategies in advance and described this as more important than uniformity in action. To further understand this phenomenon could be a subject for further research.

The level of analytic reasoning depended on the phase in the clinical reasoning process. The first two phases, *consider the situation* and *collect cues*, are by nature non-analytic [15]. However, the phase *identify problems* was the briefest, which can be explained by the specific MCI scenario. As the participants’ assignment was to perform a primary triage and perform first aid for the injured, they did not prioritise taking time and effort to identify and reflect on information to form new conclusions but were focused on working efficiently to fulfill their mission. In an MCI setting, this behaviour could be a necessity. The last phase of the clinical reasoning cycle was naturally the widest ranging, as that phase is about looking back at the experience and reflecting on the process and new learning. The participants expressed appreciation for being able to take time to reflect, as they believed this would help them to become better prepared for the future. This insight suggests that educators might benefit from emphasizing reflective practices in training sessions, helping learners to develop more comprehensive clinical reasoning skills that balance both non-analytic and analytic approaches. Further research could explore how this balance influences performance and outcomes in various clinical settings, thereby contributing to a broader understanding of clinical reasoning.

The participants emphatically spoke about their need for this type of training. All the experiences together became a learning activity, both the VR simulation itself and the interview afterward, when they were encouraged to verbalise their clinical reasoning and reflect on action [7]. The VR simulation of an MCI was described to be a suitable way to practice behaviours and to become better skilled and prepared for real-life MCIs, and the reflection afterward was valuable for obtaining better insight, self-awareness, and apprehension. Encouraging reasoning and considering all information available before generating a conclusion could lead to more analytical reasoning for ACs, regardless of clinical experience [15]. This suggests that integrating such reflective practices into training programs could enhance the overall quality of clinical decision-making. Further research could investigate how these training methodologies impact long-term competency and effectiveness in various clinical environments, contributing to a broader scientific understanding of optimal training practices in healthcare.

More knowledge is needed on whether experiential learning and reflection on action can be carried out in other ways with VR simulation. It is unclear whether ACs improve their management of MCIs after engaging in clinical reasoning after a VR simulation. There is a need for further research that can help ACs become better prepared to succeed in MCIs in the future.

### Methodological considerations

To our knowledge, this is the first study using the chart-stimulated recall technique [16], when interviewing to explore clinical reasoning after a VR simulation of an MCI. One can argue that reflecting while looking at and pausing a motion picture video is not the same thing as reflecting on a patient chart [16]. However, the method was found to be usable when interviewing as it became integrated with reflection on action and with describing ACs’ clinical reasoning about an MCI and shows that the chart-stimulated recall technique can be applicable in diverse ways.

The findings from this study are limited in that they reflect the experiences and views of a small group of homogeneous ACs. The fact that all the participants in this study were considered senior ACs and were working in special ambulance units may have affected the richness of the results of their clinical reasoning both positively and negatively. Moreover, the transferability of the findings to other participants and settings may have been affected by local recruitment [24] but goals for the ambulance service concerning how to handle an MCI are more or less equivalent worldwide. Regardless, this study has described how senior ACs conducted clinical reasoning when simulating an MCI with VR, which is an unstudied research subject and may add knowledge to other settings.

### Reflexivity

The first, second, and last authors had contextual pre-understanding prior to conducting this study, as they have a background as ACs. This may have influenced the understanding of the data, as it helped them understand the nuances when analysing and interpreting results. The authors aimed to maintain a critical approach and a journal with field notes was written during the research process to keep notes of reflection on the process and the researchers’ role and influence [24, 25]. To avoid possible pre-understanding bias problems, the third author, with no experience working in the ambulance service, reviewed the data and results iteratively [25]. However, as this study is based on a learning activity, pre-understanding could be an advantage as it improves the understanding of who the learners are, their expectations and goals and is considered critically important in simulation design and distribution [26].

## Conclusion

This study describes ACs’ clinical reasoning when simulating an MCI using VR. The findings show clinical reasoning related to the work tasks of an MCI. Non-analytic reasoning predominated when work tasks followed specific clinical guidelines, but analytical reasoning appeared when the guidelines were unclear or non-existent. Using VR simulation led to training and reflection on action in a safe and systematic way and increased their experience of self-awareness among the ACs regarding their preparedness for MCIs. Structured use of the clinical reasoning process in teaching and learning could be a useful routine to implement when learning to manage complex clinical situations, such as MCIs. Hence, simulation training with VR has the potential to be a tool for ACs to practice clinical reasoning and could improve this important competence, thereby having an impact on patient safety and outcome. This study both increases knowledge regarding ACs’ clinical reasoning in MCIs and provides insights regarding the trending use of VR for simulation training.

## Supplementary Information

Supplementary Material 1.

Supplementary Material 2.

## Data Availability

The datasets used and analysed during the current study are available from the corresponding author upon reasonable request.

## Abbreviations

MCI: Mass casualty incident
AC: Ambulance clinician
VR: Virtual reality
EMT: Emergency medical technicians
