# Supplementary material for: Exploring ambulance clinicians’ clinical reasoning when training mass casualty incidents using virtual reality: a qualitative study
Source: Scand J Trauma Resusc Emerg Med. 2024 Sep 16;32:90. doi: 10.1186/s13049-024-01255-5 (PMC11403774; doi:10.1186/s13049-024-01255-5)
Supplement: Supplementary file 2 — Supplementary Material 2. [file 13049_2024_1255_MOESM2_ESM.docx]

One of the reviewers asked for pictures/videos. However, as the university's computers were hacked, the photos are not available anymore. What still remains, is an informational video we made. This is a link to the video on YouTube, showing images from the data collection process of this article. Feel free to use parts of it, as a photo or motion picture, but you must remove the sound and the sign of the university.

Kind regards, Sara Heldring

<https://youtu.be/go8dcko0Ll8>
